# Terahertz binary computing in a coupled toroidal metasurface

**DOI:** 10.1038/s41598-024-59069-5

**Published:** 2024-04-15

**Authors:** Angana Bhattacharya, Bhagwat Singh Chouhan, Kajal Sharma, Sami M. Halawani, Amir Ahmad, Gagan Kumar

**Affiliations:** 1 Department of Physics, Indian Institute of Technology Guwahati, Guwahati, Assam 781039 India; 2 Faculty of Information Technology, King Abdulaziz University, Jeddah, Saudi Arabia; 3 College of Information Technlogy, United Arab Emirates University, Al Ain, United Arab Emirates

**Keywords:** Optics and photonics, Physics

## Abstract

The applications of terahertz metamaterials are being actively explored in recent times for applications in high-speed communication devices, miniature photonic circuits, and bio-chemical devices because of their wide advantages. The toroidal resonance, a new type of metasurface resonance, has been examined with great interest to utilize its properties in terahertz metasurface applications. This study reports a proof of concept design of a toroidal metasurface that experimentally demonstrates binary computing operations in the terahertz frequency regime. The analog computing of binary operations is achieved by the passive tuning of distance between the split ring resonators comprising the meta-molecule. The amplitude modulation is utilized as a method of determining the Boolean logic outputs of the system. The proposed metasurface could be further optimized for high amplitude modulations and active logic gate operations using tunable materials including graphene and ITO. The proposed metasurface consists of three split-ring resonators, and the near-field coupling between the adjacent resonators dictates the Boolean operations. A multipole analysis of the scattered powers of terahertz radiation determines the toroidal excitation in the metasurface. The proposed metasurfaces experimentally define AND Boolean logic operation at 0.89 terahertz, and OR Boolean logic operation at 0.97 terahertz. Numerical simulations support the experimentally obtained results. Additionally, we numerically report the excitation of NAND operation at 0.87 THz. Such toroidal analog computing metasurfaces could find applications in digitized terahertz circuits and integrated photonic devices.

## Introduction

Metamaterial (MM) technology has seen an expanding interest, as well as advancements in the last decade because of its numerous applications^1^. MMs are artificially designed structures that consist of arrays of resonant elements, termed as meta-atoms, which mimic the properties of natural materials^2^. The dimensions of the meta-atoms determine the properties of the MM. The meta-atoms are designed smaller than the wavelength of the incident radiation, and hence the non-uniformity of the meta-atom array is lost to the incident radiation resulting in a uniform new artificial material, or metamaterial. The metamaterials designed in 2-D planar surfaces are termed as metasurfaces. The meta-atoms are often in the form of resonant elements termed as split ring resonators. Metallic as well as dielectric metasurfaces have found wide applications in the design of modulators, switches, lenses etc.^2–7^. A pre-dominant application of metasurface is in the field of terahertz (THz) devices^8^. The terahertz radiation is a segment of the electromagnetic spectrum which is sandwiched between the microwave and infra-red frequencies^9^. Lying at the intersection of optics and photonics technologies, it had remained underutilized due to the lack of natural sources vibrating at THz frequencies^10^. However, MMs, with carefully designed meta-atoms, could exhibit the required terahertz responses and be utilized for various purposes. Researchers have demonstrated numerous applications of THz metasurfaces including electromagnetically induced transparency, terahertz bio-sensors, meta-modulators, filters etc^8^. THz metamaterials have also been designed in recent times to study an interesting electromagnetic resonance, termed as the toroidal dipole resonance^11^. The toroidal dipole excitation takes place when magnetic moments are aligned in a head-to-tail manner^12^. The toroidal excitation is not evident in natural materials due to the domineering effect of electric and magnetic dipole moments^13^. However, meta-atoms with toroidal symmetries could be designed such that the toroidal dipole excitation demonstrates significant effect in metasurfaces^14,15^. The inherent curiosity to study this new type of resonance has resulted in diverse examinations of the properties of toroidal metasurfaces^16–24^. Toroidal excitation has demonstrated similar far-field radiation pattern to electric dipole radiation and the interference between the two has demonstrated a non-radiating anapole configuration^25^. Further, toroidal excitation has displayed evidence of sharp resonances with high quality factors^19,26–28^. Active tuning of metasurfaces, dual toroidal resonances, toroidal BICs have seen wide interest^19,26^. There is a rising curiosity in the applications and capacities of the toroidal resonance in THz metasurfaces. Considering these aspects, this article studies the analog computing capabilities in a toroidal THz metasurface. Boolean operations are the basic building blocks of computational circuits. Logic gates make decisions based on a combination of inputs fed into it, with both inputs and outputs in the form of Boolean signals^29^. The Boolean operations are performed using light in optical logic gates^30^ . A reconfigurable MEMS Fano resonant metasurface has been demonstrated that performs terahertz logic gate operations with electrical inputs exhibiting XOR and XNOR operations at far field, and NAND operation in the near-field^31^. Further, NOR and OR Boolean operations have been demonstrated in an all-optical terahertz logic gate made from a semiconductor-metal hybrid metasurface^32^. THz logic gates have also been demonstrated in metasurfaces based on spoof plasmons, in electro-thermally tunable metasurfaces, and in graphene-based metasurfaces^33–36^. However, toroidal metasurfaces have not been explored to harness their Boolean operation capabilities. The study of Boolean computing in a toroidal THz metasurface, similar to analog computation, provides an extra degree of freedom to explore the novel toroidal excitation for computational applications in photonic devices.

In this study, we report the computation of Boolean operations in a toroidal metasurface by studying its transmission response in the terahertz regime. The carefully designed metasurface consists of three split ring resonators, and the near field coupling between the resonators determines the Boolean states 0 (indicating OFF state) and 1 (indicating ON state) in the metasurface. A thorough understanding of the logic gate operations is provided by experimental results and supported by numerical simulations. The proposed geometry demonstrates low amplitude modulation among the Boolean states, but the design can be further optimized to show higher amplitude modulations for the different Boolean states by incorporating asymmetries and exciting Fano or BIC-type resonances. The proposed design serves as an experimental proof of concept system to demonstrate the use of toroidal resonances for applications in actively tunable logic gates, in edge detection, and image processing metasurfaces. Further, the idea can be extended to other frequency domains by tweaking the toroidal metasurface geometry. The article is arranged as follows. We initially discuss the numerical designing of the metasurfaces, their experimental fabrication, and THz characterization. Following this, the excitation of the toroidal resonance in the metasurface is discussed via surface current profiles and a multipole analsysis of scattered power. This is followed by the analysis and discussion of Boolean operations in the metasurface both experimentally and numerically. The conclusion section provides a summary of the experimental and numerical findings of the study, the associated drawbacks, scope of improvements, and future prospects of the study.

## Metamaterial design and simulation

**Figure 1:**
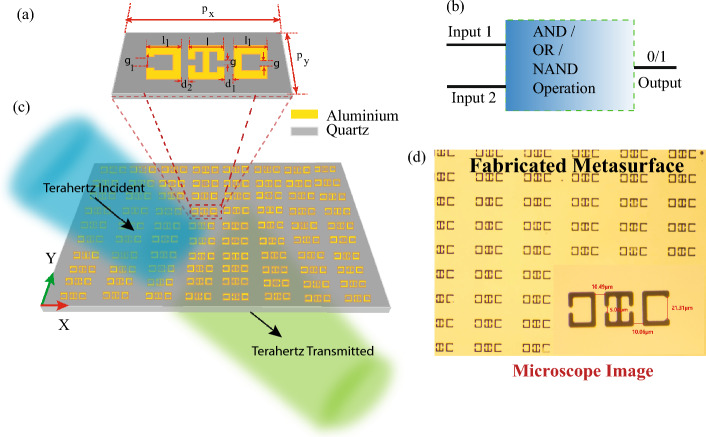
(**a**) Schematic of the proposed meta-atom having periodicity along x and y direction as “$p_{x}$” and “ $p_{y}$” respectively, length of resonator “ l”, capacitive gap “ g”, and distance between adjacent resonators “ $d_{1}$”, and “$d_{2}$”. (**b**) A schematic of the two-input Boolean computations in the MM. (**c**) A schematic of the metasurface array. Y-polarized THz radiation is incident normally on the metasurface. (**d**) Microscope image of the fabricated metasurface array. A single meta-atom is shown in a magnified view.

The basic design of the metasurface was inspired by a previously studied MM geometry, and consists of three aluminum meta-atoms in a quartz substrate [22]. However, adequate modifications have been made keeping in mind the applications of the metasurface. Figure 1a shows the schematic of a meta-atom of the proposed metasurface. The design was chosen for its simplicity and ease of fabrication. The central split ring resonator (SRR) demonstrates toroidal nature while the left and right C resonator shows a dipolar nature. The periodicity along the x direction is “ $p_x$”= 200 $\upmu$m, while the periodicity along the y direction is “ $p_y$”= 100 $\upmu$m. The length of the central resonator was chosen as “ l”=35 $\upmu$m and that of the C resonators was “ $l_1$”=32 $\upmu$m. The width of the resonators was 5 $\upmu$m. The capacitive gaps on the central resonators “ g” is 5 $\upmu$m. The capacitive gap on the right resonator is g =5 $\upmu$m. The capacitive gap on the left resonator is, “$g_1$”= 21$\upmu$m. The breadth of all the SRRs is, “ b” = 35 $\upmu$m. The distance between the central resonator and C-shaped resonator on the right-hand side is termed as “ $d_1$” and that between the central and left “ C ” resonator is termed as “ $d_{2}$”. The variation of $d_1$ and $d_2$ is analyzed to study the logic gate operation in our MM sample. The design of the metasurface and numerical simulations were performed using CST Microwave Studio simulation software. Figure 1c shows the schematic of the metasurface array on which terahertz (THz) radiation is incident normally, with polarization parallel to the capacitive gaps. Figure 1b demonstrates a block diagram of the logic gate operations that could be undertaken via the MM. A 2-input logic gate provides a Boolean output on the basis of the combination of signals fed in the input. Figure 1d shows a microscope image of the experimentally fabricated metasurface using photolithography in a clean room environment. The image is in a mirror image format as observed under the microscope. The image of a single meta-atom is shown in a magnified view for better clarity and understanding.

## Fabrication and characterization of the terahertz metasurface

The metasurface was fabricated using traditional photolithography technique. A quartz substrate ($\varepsilon =3.7$) was initially cleaned using acetone and further, coated with a 200 nm thick aluminium layer using thermal deposition technique. Further, it was spincoated with positive photoresist (S1813) and the desired pattern was transferred to the quartz substrate using UV radiation and a hard mask. The sample was then developed and the 1 cm-by-1 cm array of meta-atoms were obtained. The final metasurface configuration was fabricated for four different geometries for values of of $d_1$ and $d_2$ corresponding to (10 $\upmu$m, 10 $\upmu$m), (10 $\upmu$m, 20 $\upmu$m), (20 $\upmu$m, 10 $\upmu$m), and (20 $\upmu$m, 20 $\upmu$m) respectively. The fabricated samples were then characterized using a THz-time domain spectroscopy (THz-TDS) setup consisting of two fibre-coupled photo-conductive antennas^37^. The response of the bare quartz substrate, termed as reference, is first analyzed followed by analyzing the response of the metasurface (sample). The ratio of the sample response to the reference response provides the transmission through the metasurface.The Fabry-Perot (F-P) effect is prominently observed in THz-TDS measurements due to multiple reflections from the substrate before the THz signal reaches the detector. The effect displays itself as wobbles in the spectra. To eliminate this effect, the secondary peaks of the time-domain data is ignored and the Fourier transform is taken for the first peak of the time-domain data. This ensures that the obtained frequency domain data has minimum F-P influence.

## Results and discussion

**Figure 2:**
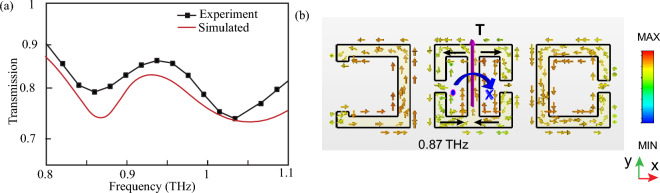
(**a**) Simulated and experimentally measured terahertz transmission response of the metasurface for the $d_1$ = $d_2$ = 10 $\upmu$m configuration. (**b**) Surface current profile of the metasurface for the resonance at 0.87 THz. Toroidal dipole excitation is shown along the Y-axis.

The THz response of the metasurface is studied via simulations, as well as experimental measurements of the transmission spectrum. The response is measured for a configuration where the distance between the adjacent resonators is $d_1$ = $d_2$ = 10 $\upmu$m. The transmission spectrum is shown in Fig. 2. The red line in Fig. 2a shows the simulated transmission spectrum using CST Microwave Studio software, and the black curve in Fig. 2a illustrates the experimentally measured transmission spectrum, measured using the THZ-TDS setup. From the simulated transmission spectrum, we observe two resonance dips at 0.87 THz and 1.07 THz. The THz transmission through the fabricated metasurface is measured, and similarly demonstrates two resonances and a close match between the simulated and experimentally measured results is observed. In this study, we have focused on the first resonance at 0.87 THz. The surface current profile of the metasurface at 0.87 THz is analyzed, as shown in Fig. 2b. It may be observed that, for the central resonator, the surface current profile flows clockwise on the right-hand arm and flows anticlockwise on the left-hand arm of the resonator. The clockwise flow of current on the right arm leads to a magnetic moment going inside the plane on the right side (indicated by the cross symbol), while the anticlockwise flow on the left arm leads to magnetic moment coming outside the plane on the left side of the resonator (indicated by the dot symbol). This end-to-end formation of magnetic moments lead to the excitation of a toroidal dipole moment along the y direction. Thus, there is a clear excitation of toroidal dipole moment in the metasurface at the resonant frequency of 0.87 THz.

**Figure 3:**
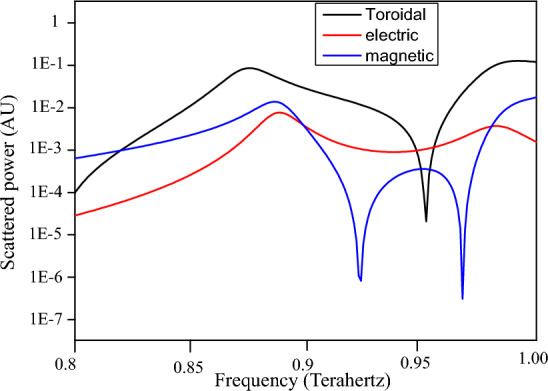
Multipole analysis of scattered power by electric, magnetic, and toroidal dipole moments over the THz frequency range.

Further, we study the nature of electromagnetic resonances in the metamaterial structure. A multipole analysis is performed over the terahertz frequency range of 0.8–1 THz and the power scattered by electric, magnetic, and toroidal dipolar excitation is plotted for the $d_1= 10\,\upmu$m, $d_2=10\,\upmu$m configuration, as shown in Fig. 3. The multipole analysis has been studied in several articles wherein the power scattered by the different electromagnetic moments have been evaluated theoretically^38,39^. The black line in Fig. 3 indicates toroidal scattered power, the blue line indicates power scattered by magnetic dipole moment, and the red line indicates electric dipole scattered power. It is seen that at 0.87 THz where the metasurface has its first resonance, there is highest toroidal scattered power. This confirms the toroidal behaviour of the metasurface. It is also observed that in the frequency range of 0.87–0.95 THz, and also in the range of 0.96–1 THz, there is highest toroidal dipole scattered power. Thus, the window containing the frequencies that demonstrate Boolean operations has pre-dominantly toroidal scattered power.

## Study of AND and OR Boolean operations

**Figure 4:**
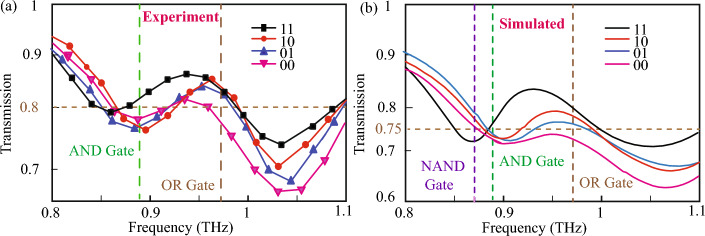
(**a**) Proof of concept of Boolean operations for various input states of the metasurface at varying frequencies. A transmission amplitude of 80% and higher is taken as the cutoff for logic output state 1 (ON state) for experimental measurements, while a cutoff of 75% is taken for simulated results. The input states: 00, 01, 10, 11 are determined by the passive displacement of the C-resonators from the central resonator. (**a**) Boolean operation at 0.89 THz (AND), and 0.97 THz (OR) for experimentally measured results. (**b**) Boolean operations at 0.87 THz (NAND), 0.89 THz (AND), and 0.97 THz (OR) for numerically simulated results.

The study of Boolean operations is done via the change in the distance between the central resonator and the two other split resonators on the side of the central resonator. The initial configuration that was analyzed had a symmetrical structure with both the C resonators at a distance $d_1=d_2=10\,\upmu$m from the central SRR. The transmission spectrum for this symmetric configuration is discussed in the previous section, and shown in Fig. 2. We consider this configuration as the Boolean input “11” configuration. The displacement of the left-side side resonator from the central resonator by a unit of $d_2=20\,\upmu$m, while the right-hand side resonator is fixed at $d_1=10\,\upmu$m, is considered as a Boolean input “ 10”. This variation in the transmission for varying the distances of the C-shaped resonators from the central resonator is shown in Fig. 4. The experimentally measured transmission spectrums for the fabricated metasurfaces of varying $d_1$,and $d_2$ are depicted in Fig. 4a. The transmission spectrum for the $``10''$ configuration is shown in Fig. 4a by the red curve. A blue shift in the first resonance dip is observed as it is shifted to 0.9 THz as compared to the “11” configuration. The configuration where the right SRR is at a distance $d_1=20\,\upmu$m from the central resonator, while $d_2$ is fixed at 10 $\upmu$m is termed as the “01” input condition. For this configuration, the resonance dips are observed at 0.89 THz and at 1.05 THz as can be seen via the blue line in Fig. 4a. Further, the configuration where both the C resonators are separated from the central resonator by distance $d_1=d_2=20\,\upmu$m is termed as the “00” configuration. We term this as the “00” input as it is believed that increasing the distance between the central resonator and the C resonators will lead to a reduced near field coupling between the SRRs. Hence, the decrease in the near field coupling is assumed to correspond to the “ OFF” state or “00” state in our study. Similarly, we believe that the $d_1=d_2=10\,\upmu$m input state is the “ ON” state, or “ 11” state as both the C-SRRs will strongly couple to the middle resonator. For the 00 configuration we observe one resonance dip at approximately 0.89 THz and a broad resonance dip at 1.05 THz as shown by the pink curve of Fig. 4a. On varying the distances $d_1$ and $d_2$, a variation in transmission amplitude and resonance width is observed. The resonances become broader and there is an increase in the transmission depth as the distances are varied. It is evident that all the resonance dips and peaks of the transmission spectrum occur within a small window of 0.8–1.1 THz. For our study of logic gate operation in a MM, we intend to utilize this narrow THz window to explore the various logic gates in the metasurface.

To realize the Boolean operations, we scrutinize the different regions that could exhibit logic gate outputs for the specified inputs for the window of 0.8–1.1 THz, as shown in Fig. 2. In this study, we fix a transmission amplitude of 80% to qualify for a Boolean output 1 state. A transmission amplitude less than 80% is assumed to be a Boolean output 0 state. The black curve in Fig. 4 indicates the input 11 state, the red curve indicates the input 10 state, the blue curve indicates 01 state, and the pink curve indicates 00 state, as discussed. It is observed that at 0.89 THz, the 11 state has a transmission higher than 80% while the 10, 01, and 00 states demonstrate a transmission amplitude of less than 80%. The truth table corresponding to this configuration at 0.89 THz is described in Table 1. The truth table output for all states besides the 11 states is Boolean 0, while the output for the 11 state is Boolean 1. From the truth table, Table 1, it can be understood that the metasurface at 0.89 THz corresponds to a Boolean AND operation. Further, the response of the metasurface at 0.97 THz is analyzed. The black, red, and blue curves corresponding to the 11, 10, and 01 input states respectively, and they all have transmission higher than 80%, while the pink curve corresponding to the 00 input state has a transmission less than 80% at 0.97 THz as can be seen in Fig. 4. The corresponding truth table is depicted in Table 2. The output of the truth table at 0.97 THz corresponds to the OR Boolean output configuration. Thus, the metasurface experimentally demonstrates AND, and OR Boolean operations at 0.89 THz and 0.97 THz respectively.

**Table 1:** Truth table for AND Boolean operation at 0.89 THz.

Input 1 ($d_{1}$)	Input 2 ($d_{2}$)	Output
0	0	0
0	1	0
1	0	0
1	1	1

**Table 2:** Truth table for OR Boolean operation at 0.97 THz.

Input 1 ($d_{1}$)	Input 2 ($d_{2}$)	Output
0	0	0
0	1	1
1	0	1
1	1	1

The experimental results are further verified using numerical simulations by studying the transmission spectrum of the metasurface for the different configurations of $d_1$ and $d_2$. The simulation results are depicted in Fig. 4b. The black curve in Fig. 4b illustrates the (1,1) configuration. The first resonance for the black curve lies at 0.87 THz and the second resonance dip is at approximately 1.05 THz. Further, the transmission spectrum corresponding to the other variations of $d_1$ and $d_2$ are also analyzed. The red curve in Fig. 4b corresponds to the simulated (1,0) configuration. The blue curve corresponds to the simulated (0,1) configuration, while the pink curve in Fig. 4b corresponds to the simulated (0,0) configuration. It may be observed that there is some small mismatch between the amplitude of the simulated transmission dips as compared to that of the experimentally measured curves depicted in Fig. 4a. These differences in the amplitude of the transmission dips may be attributed to the fabrication imperfections of the metasurfaces, environmental effects, diffraction losses, and due to the difference in the resolution of the CST-simulations and measured results using the THz-TDS setup. To take into account the shift in the transmission amplitude for the simulation results, a cutoff of 75% transmission is set for the Boolean output 1 in the simulated results. From Fig. 4b, it can be observed that at 0.89 THz, the black curve corresponding to Boolean input (1,1) is higher than the cutoff transmission, leading to a Boolean output of 1. The other curves corresponding to input configurations (1,0), (0,1), and (0,0) are below the cut-off transmission of 75%, hence leading to a Boolean output of 0. The truth table for the simulated curve at 0.89 THz matches with the truth table in Table 1 for AND Boolean operation, illustrated for the experimentally measured results at 0.89 THz. Thus numerically, AND operation is obtained at 0.89 THz. Next, the response at 0.97 THz is analyzed, and it is observed that the simulated curves have transmission higher than the 75% cutoff for all input configurations, corresponding to a Boolean output 1, except the (0,0) configuration which corresponds to a Boolean output 0. This is matched with the truth table for OR gate as depicted in Table 2. Thus, at 0.97 THz, simulation results match with the experimental results, and OR operation is obtained. Hence, the metasurface demonstrates AND and OR Boolean operations in the THz domain.

### NAND operation

Further, for the simulated transmission spectrum, the response at 0.87 THz was analyzed and it was observed that transmission is higher than the cutoff value of 75% for the (01), (10), and (0,0) configurations, while it is lower than the cutoff for the (1,1) configuration. The truth table corresponding to this observation is shown in Table 3. The response at 0.87 THz corresponds to a Boolean output of NAND configuration. Thus, via simulation, the metasurface demonstrates OR, AND, and NAND operations at 0.97 THz, 0.89 THz, and 0.87 THz respectively.

**Table 3:** Truth table for NAND Boolean operation at 0.87 THz.

Input 1 ($d_{1}$)	Input 2 ($d_{2}$)	Output
0	0	1
0	1	1
1	0	1
1	1	0

**Figure 5:**
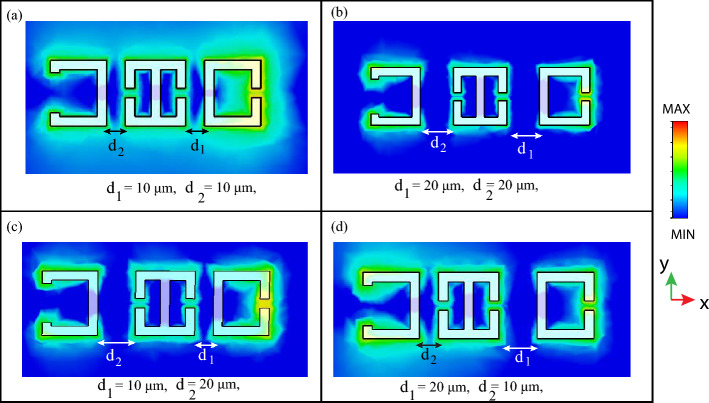
The electric field excited in the four metasurface configurations and analysis of their near field coupling. (**a**) Electric field profile for the $d_1=d_2=10\,\upmu$m metasurface configuration. (**b**) Electric field profile for the $d_1=d_2=20\,\upmu$m metasurface configuration. (**c**) Electric field profile for the $d_1=10\,\upmu$m $d_2=20\,\upmu$m metasurface configuration. (**d**) Electric field profile for the $d_1=20\,\upmu$m, $d_2=10\,\upmu$m metasurface configuration.

The truth tables for the experimentally analyzed Boolean operations at 0.89 THz, 0.97 THz, and 0.87 THz are illustrated below. The two inputs, input 1 and input 2, depict the distance $d_1$, and $d_2$, with the input being “ high” or “1 ” for $d_1$ or $d_2$ = 10 $\upmu$m, and the input being “ low” or “ 0” for $d_1$ or $d_2$ = 20 $\upmu$m. The near-field coupling between the resonators was also studied for the different metasurface configurations at the first resonance. Figure 5 demonstrates the near field coupling between the resonators for varying distances $d_1$ and $d_2$. Figure 5a shows the $d_1=d_2=10\,\upmu$m configuration, and it may be observed that all three resonators are excited and there is strong near-field coupling between the SRRs which verify our consideration of the $d_1=d_2=10\,\upmu$m configuration as the 11 Boolean input. We next studied the electric field coupling for the $d_1=d_2=20\,\upmu$m configuration. We observe that the three resonators are individually excited by the y-polarized THz radiation, however there is reduced near field coupling between the resonators, as can be seen from Fig. 5b, and we believe this reduced coupling validates our consideration of the $d_1=d_2=20\,\upmu$m configuration as the 00 Boolean input. The interaction between the SRRs for $d_1= 10\,\upmu$m, $d_2=20\,\upmu$m configuration is shown in Fig. 5c. It may be observed that there is stronger near field confinement between the central and right resonators, and weak interaction between the central and left resonators and thus, we consider the $d_1= 10\,\upmu$m, $d_2=20\,\upmu$m configuration as the 10 Boolean input. The near field interaction in the $d_1= 20\,\upmu$m, $d_2=10\,\upmu$m configuration is shown in Fig. 5d, and similar to the case of Fig. 5c, stronger near field interaction is observed between the central and left resonator as compared to the central and right resonator. Thus, this verifies our consideration of the $d_1= 20\,\upmu$m, $d_2=10\,\upmu$m configuration as the Boolean 01 input.

## Conclusions

This study explores the design of a toroidal metasurface demonstrating Boolean operations in the range of 0.85 THz to 1 THz. It is confirmed via electric field profiles that there is strong bright-bright mode coupling between the SRRs when they are placed close together at a distance of $d_1=d_2=10\,\upmu$m, which is assumed to be the “11” Boolean input state. Further, the reduced coupled state corresponding to $d_1=d_2=20\,\upmu$m is taken as the “00” input state. The variation of distances $d_1$ and $d_2$ leads to shifts in the transmission spectrum and the displacements are analyzed to demonstrate Boolean operations at different frequencies. We report the excitation of AND and OR Boolean operations experimentally and numerically in the metasurface at 0.89 THz and 0.97 THz respectively. Further, numerical simulations demonstrate NAND Boolean operation at 0.87 THz. The numerical and experimental measurements show a close match in the results. The near field coupling between the resonators is also analyzed to explain the Boolean computations corresponding to the geometry of the resonators. Further, a multipole analysis is performed over the frequency range of 0.8–1 THz and toroidal behaviour at the resonance is confirmed for the metasurface. The study serves as a proof of concept idea to demonstrate and further improve the functioning of toroidal devices in the terahertz domain for analog computing, edge detection, and logic gate operations. There is further scope in the study via active modulation of parameters to demonstrate an active toroidal logic gate in the terahertz region. The limitation of low amplitude modulation for the Boolean ON and OFF states could be overcome by optimized metasurface designs having sharper toroidal resonance with increased transmission depth. Further, the passive nature of the Boolean computations could be improved by introducing an active material to demonstrate active toroidal Boolean computations. Such toroidal metasurfaces could be significant in the design of futuristic terahertz compact devices and on-chip photonic circuits.

## Data Availability

The data that support the findings of this study are available from the corresponding author, A.A, upon reasonable request.
